# The geometric phase controls ultracold chemistry

**DOI:** 10.1038/ncomms8918

**Published:** 2015-07-30

**Authors:** B. K. Kendrick, Jisha Hazra, N. Balakrishnan

**Affiliations:** 1 Theoretical Division (T-1, MS B221), Los Alamos National Laboratory, Los Alamos, New Mexico 87545, USA; 2 Department of Chemistry, University of Nevada, Las Vegas, Nevada 89154, USA

## Abstract

The geometric phase is shown to control the outcome of an ultracold chemical reaction. The control is a direct consequence of the sign change on the interference term between two scattering pathways (direct and looping), which contribute to the reactive collision process in the presence of a conical intersection (point of degeneracy between two Born–Oppenheimer electronic potential energy surfaces). The unique properties of the ultracold energy regime lead to an effective quantization of the scattering phase shift enabling maximum constructive or destructive interference between the two pathways. By taking the O+OH→H+O_2_ reaction as an illustrative example, it is shown that inclusion of the geometric phase modifies ultracold reaction rates by nearly two orders of magnitude. Interesting experimental control possibilities include the application of external electric and magnetic fields that might be used to exploit the geometric phase effect reported here and experimentally switch on or off the reactivity.

## Abstract (web summary)

Ultracold reactions can give insights into reactions dynamics in the quantum regime. Here, the authors show that the geometric phase can have a dramatic effect on ultracold reactions, enhancing or suppressing rates by nearly two orders of magnitude in the reaction studied.

## Graphical abstract

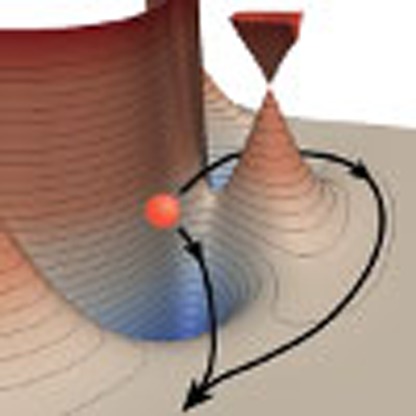

The geometric phase (GP) effect in molecules refers to the sign change associated with the Born–Oppenheimer adiabatic electronic wave function (typically the ground state) when transported along a closed path encircling a conical intersection (CI), a point of degeneracy between two electronic potential energy surfaces (PESs). The sign change was noticed by Longuet–Higgins^1^ and Herzberg–Longuet–Higgins^2^ over 56 years ago in the context of molecular spectra. In order for the total Born–Oppenheimer molecular wave function to remain single valued, the electronic sign change implies that a corresponding sign change must also occur on the nuclear motion wave function. This sign change occurs even when the energy of the nuclear motion lies well below the energy of the CI and the nuclear motion is confined to just one adiabatic electronic PES. The implications of the sign change for chemical reactions occurring on a single Born–Oppenheimer electronic PES including the effects of identical nuclei was treated by Mead and Truhlar^3^ and Mead^4^,^5^,^6^. They showed that a consistent treatment of the sign change and identical nuclei leads to a generalized Schrödinger equation for the nuclear motion that contains a vector potential interaction analogous to that of a magnetic solenoid centered at the CI^3^. Berry^7^ later showed that the appearance of a vector potential and its associated GP (also known as the Berry phase) is a general consequence of the adiabatic transport of a quantum state^8^. Mead originally referred to this effect as the ‘Molecular Aharonov–Bohm' (MAB) effect^4^, and showed that the interference term between the reactive and non-reactive contributions to the scattering cross-section for the fundamental H+H_2_ reaction changes sign when the GP or MAB effect is included^5^. However, the experimental detection of Mead's predictions has been elusive to this day despite heroic experimental efforts^9^. Numerous theoretical scattering calculations for the H+H_2_ system and its isotopic variants that include the GP have also been performed beginning with the work of Lepetit and Kuppermann^10^. Other molecular systems have also been treated^11^,^12^,^13^. Surprisingly, for the lower-symmetry purely reactive isotopic reactions H+D_2_→D+HD and D+H_2_→H+HD, it was shown that the GP effects seen in both the partial integral and differential cross-sections all but cancel out when the partial cross-sections are summed over all values of total angular momentum *J* to obtain fully converged cross-sections^14^,^15^,^16^,^17^. A similar cancellation also occurs in the H+H_2_ reaction for transitions that involve only reactive contributions to the cross-sections^17^. However, for the transitions that involve *both* reactive and non-reactive contributions, the cancellation does not occur and Mead's original prediction of GP effects for this case were confirmed^17^. The surprising cancellation of the GP effects for the purely reactive transitions with respect to the partial wave sum was later confirmed by Juanes–Marcos *et al*.^18^. Furthermore, by considering direct and looping contributions to the reactive scattering amplitude, it was shown that they scatter into distinctly different angular regions^19^. Consequently, very little interference occurs between the two pathways resulting in essentially no observable GP effects in both the differential and integral cross-sections^19^. At higher collision energies and larger values of total angular momentum *J*, some interference is observed but it results in rapid oscillations of the differential cross-section as a function of scattering angle^19^. These rapid oscillations average out and vanish in the integral cross-sections^19^,^20^. Therefore, the most promising experimental detection of GP effects to date has focused on the elusive small-amplitude interference oscillations in the differential cross-sections at high collision energies below the CI^9^. Theoretical calculations have also been performed for energies above the energy of the CI^21^,^22^. These calculations show large bi-modal structures in the DCS because of the GP but no significant effects were seen in the integral cross-sections.

As discussed above, all previous experimental and theoretical studies of GP effects in chemical reactions have been performed at thermal energies. Remarkable experimental progress in recent years in cooling and trapping molecules at cold (<1 K) and ultracold (<1 mK) temperatures has opened up an entirely new energy regime^23^,^24^,^25^,^26^. At ultracold collision energies chemistry often becomes quantum-enhanced due in part to the very large de Broglie wavelengths, quantum tunnelling and quantum threshold effects. In the ultracold limit, the rate coefficients obey the Bethe–Wigner threshold laws^27^,^28^ and approach a finite non-zero value in the zero temperature limit. For many reactions, the ultracold rate coefficient can often be larger than its value at thermal energies. A natural question to ask is ‘Can GP effects become enhanced at ultracold collision energies?'. At ultracold collision energies only a single partial wave (s-wave) contributes to the reaction so that the cancellation of GP effects over the partial wave sum cannot occur as it does at thermal energies and the scattering is isotropic. In contrast to the situation discussed above for H+H_2_ at thermal energies, the direct and looping contributions to the cross-sections scatter into the *same* angular region with the potential for significant interference. For reactive collisions in which the direct and looping contributions are of similar magnitude, the interference term (depending on its sign and magnitude) can effectively turn on or turn off the reaction entirely. Since the GP changes the sign of the interference term between the direct and looping contributions, it can effectively control the reaction.

We report here theoretical predictions of GP effects in ultracold chemical reactions. Specifically, large GP effects are predicted to occur in ultracold chemical reactions, for which: (a) the relevant Born–Oppenheimer adiabatic electronic PES exhibits a CI, (b) the direct and looping contributions to the scattering amplitude are of similar magnitude and (c) their phases are quantized (or nearly so). These three conditions are readily met in the experimentally relevant O+OH (*v*=0, *j*=0)→H+O_2_ (*v*′, *j*′) reaction, which will serve as our prototypical system. For this reaction, it is shown that the GP can enhance or suppress the rotationally resolved reaction rates by nearly two orders of magnitude. The OH radical has been cooled and trapped using the buffer gas and Stark decelerator techniques^29^,^30^,^31^, and successful experiments on evaporative cooling of OH have recently been carried out^32^. Numerous experimental and theoretical studies of collisions involving OH molecules in the cold and ultracold temperature regimes have been reported in recent years, and the O+OH reaction continues to be the topic of considerable experimental and theoretical investigations^33^,^34^,^35^,^36^,^37^,^38^,^39^,^40^.

## Results

### The interference mechanism

**Figure 1:**
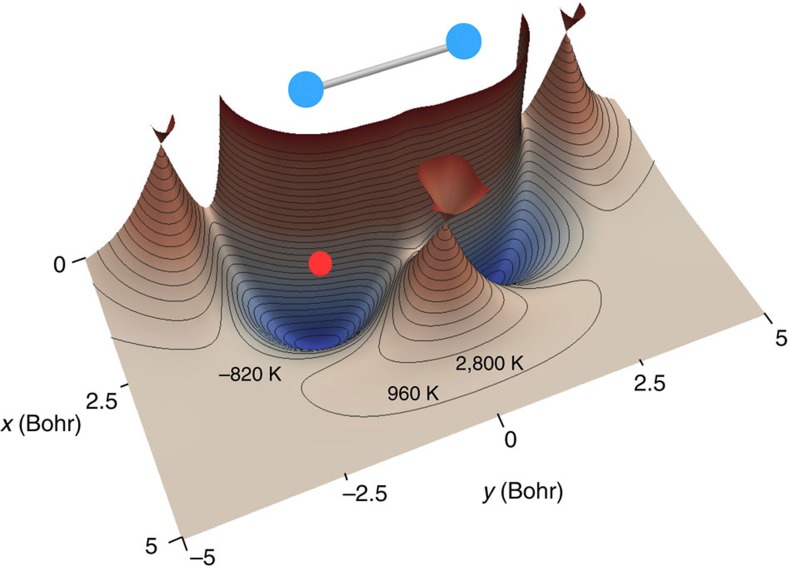
HO_2_ potential energy surface. A 2D slice of the Born–Oppenheimer PES for the ^2^*A*″ ground electronic state of HO_2_ is plotted in the product region for a fixed O_2_ (blue spheres) separation of 2.28 bohr. The potential energy is relative to the bottom of the asymptotic H+O_2_ well and is plotted as a function of the *xy* location of the hydrogen (red sphere) relative to the centre of the O_2_ bond. The prominent deep potential wells (dark blue region) correspond to the bound HO_2_ molecule. Three CIs are readily visible: two are located at linear geometries *x*=0, *y*=±3.5 (bohr) and a third is located at a T-shaped (*C*_2*v*_) geometry *x*=2.65, *y*=0 (bohr). The upper cones for each CI correspond to the excited electronic state (to aid visualization, a cut plane was used at 34,800 K so that the potential surfaces above this energy are not plotted). The contour lines are uniformly spaced at intervals of 1,800 K.

**Figure 2:**
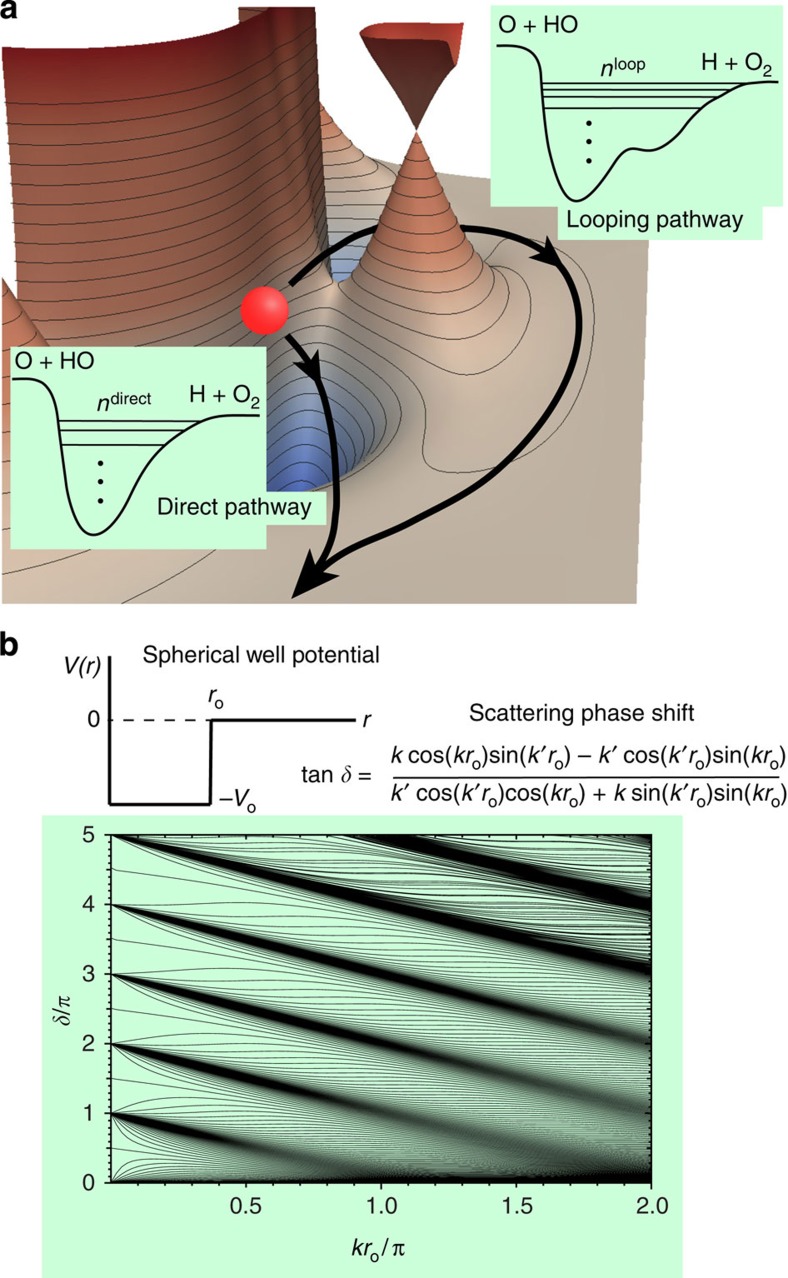
Interference between the direct and looping pathways. The direct and looping pathways around the *C*_2*v*_ CI for H–O_2_ are sketched in **a**. The scattering amplitudes for each of these pathways are summed to obtain the total scattering amplitude for the reactive collision. The reactivity can be enhanced or suppressed if the two scattering amplitudes interfere constructively or destructively, respectively. The inset figures in **a** show the effective potentials along each pathway, which support a different number of bound states. (**b**) plots the theoretically derived phase shift *δ*/*π* for *s*-wave (*l*=o) scattering of a particle of mass *μ* from a model spherical well potential with a depth of *V*_o_=*q*^2^/(2*μ*) and radius *r*_o_. Each curve corresponds to a different well depth separated by *q*=0.025 *π*/*r*_o_ where *k*′^2^−*q*^2^=*k*^2^. The notable feature in this plot is that the phase shift becomes effectively quantized in the ultracold limit *k*→0.

A two-dimensional (2D) slice of the three-dimensional electronically adiabatic ground state ^2^*A*′′ PES for the HO_2_ molecule is plotted in Fig. 1. Most notable are the two deep symmetric wells (≈−26,000 K relative to the bottom of the H+O_2_ asymptote), which correspond to the bound HO_2_ complex. Three CIs are also clearly visible: one occurs for T-shaped or *C*_2*v*_ geometries (that is, O–H–O) and the other two occur at collinear geometries (that is, H–O–O and O–O–H). In the present treatment, we consider GP effects due to the *C*_2*v*_ CI. The scattering methodology for including GP effects due to collinear CIs is under development^41^. The reaction is exoergic (by ∼8,100 K) and proceeds along a barrierless reaction path through the deep potential well. The total available energy for the ultracold collision is that of the reactant OH (*v*=0, *j*=0) state, which is ≈9,400 K relative to the bottom of the asymptotic product H+O_2_ well. The minimum energy of the *C*_2*v*_ CI lies at ≈22,000 K and the maximum energy encountered along the minimum energy pathway (MEP) encircling the *C*_2*v*_ CI is ≈580 K (ref. 42). Thus, even though the ultracold collision of O+OH initiates with a tiny kinetic energy (1 μK), the total energy (≈9,400 K) is much larger than the maximum energy encountered along the MEP around the CI (≈580 K). Thus, the CI can be easily encircled, and the direct and looping contributions to the scattering amplitude can have similar magnitudes (see Fig. 2). In general, the encirclement of a CI at ultracold collision energies does not require the reaction to be exoergic (although that is often a favourable situation). Endoergic reactions as well as reactions with an entrance barrier can still exhibit favourable encirclement of a CI at ultracold collision energies, provided the reactant state is in a suitably high vibrationally and/or rotationally excited state (and with total energy still well below the energy of the CI).

The total scattering amplitude can be decomposed as 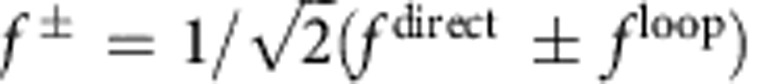 where 
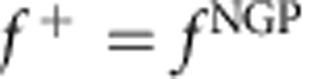
 and 
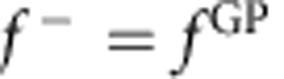
 (refs 19, 43, 44, 45). The amplitudes *f*^GP^ (GP) and *f*^NGP^ (No GP) are the scattering amplitudes computed with and without including the GP, respectively. The magnitudes of *f*^direct^ and *f*^loop^ depend on the details of the scattering process. In general, the magnitudes can be similar for situations in which the maximum potential energy encountered along the MEP encircling the CI lies below the available total energy. The energetics depend on the particular reactant and product states involved and the details of the Born–Oppenheimer electronic PES for the particular molecule of interest. If the direct and looping contributions are equal in magnitude 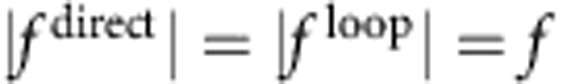 and we write the complex scattering amplitudes as 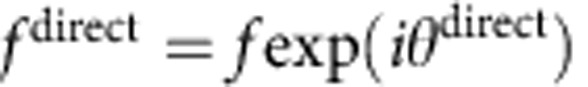 and 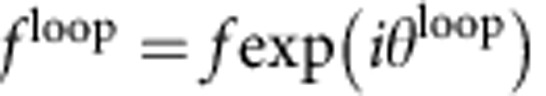, then 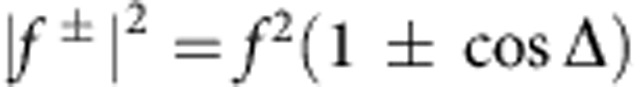 where 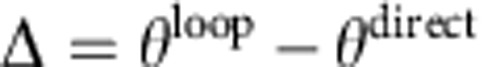 is the phase difference. Furthermore, if the phases are quantized so that the phase difference is a multiple of *π* (that is, Δ=*nπ* where *n* is an integer), then the square modulus of the scattering amplitude that gives the angular distributions will be zero or 2*f*^2^ depending on whether ±cosΔ is −1 or +1, respectively. For example, if for a particular scattering process cosΔ=1, then 
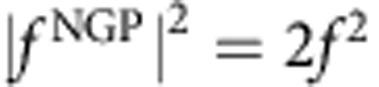
 and 
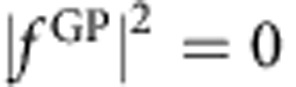
. That is, the GP completely turns off the reaction. Conversely, if cosΔ =−1, then 
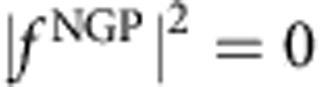
 and 
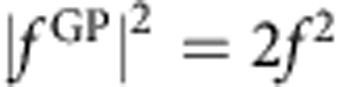
 so that the GP turns on the reaction in this case. In actual scattering situations, the phases are not exactly quantized and the scattering amplitudes are not exactly equal so that variations between the two extreme values (0 and 2*f*^2^) occur. However, the possibility of realizing very large GP effects on the chemical reaction rate is clear. In situations where the magnitudes of the *f*^direct^ and *f*^loop^ are significantly different, the square modulus of the total scattering amplitude is given by the general expression 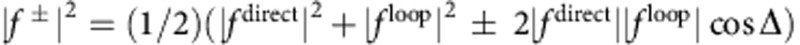. If the magnitude of one of the amplitudes is much larger than the other, then the relative magnitude of the interference term is small and 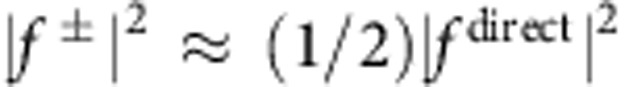 or 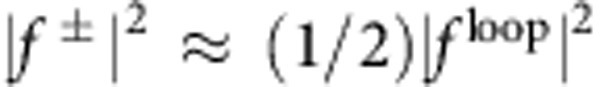 and there is little or no GP effect (that is, 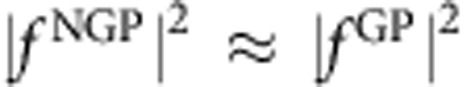).

We have shown that the ultracold reaction O+OH→H+O_2_ meets the first two criteria for realizing a large GP effect, namely (a) the PES exhibits a CI and (b) the energetics are favoruable for encirclement of the CI. The third criterion requires the quantization (or nearly so) of the phase difference between the direct and looping scattering amplitudes. The phase quantization is a unique feature of the ultracold energy regime. Figure 2 summarizes the phase quantization mechanism. In the zero energy limit, the scattering is isotropic and a spherical well can be used as a simple model of the collision process where the effective depth and width of the well corresponds to different collision pathways (and different reactant and product states). Figure 2b plots the *s*-wave scattering phase shift *δ* for a spherical potential well given by *V*(*r*)=−*V*_o_ for *r*≤*r*_o_ and *V*(*r*)=0 for *r*>*r*_o_ as a function of collision energy and well depth. In the zero energy limit (that is, wave vector *k*→0), this figure shows that the scattering phase shift becomes effectively quantized. That is, the most likely value for the phase shift at zero energy is near a multiple of *π* regardless of the well depth. As the well depth is varied for collision energies near zero, the phase shift rapidly jumps from one value of *nπ* to another. The jumps are due to resonances that occur at *δ*_*r*_=(*n*+1/2)*π* as a continuum state drops into the potential well as the well depth is increased. In fact, the *nπ* phase shift indicates the number of bound states *n* supported by the well. As sketched qualitatively in Fig. 2a, the direct and looping scattering amplitudes traverse different pathways along the PES encircling the CI as the reaction proceeds from reactants to products. The effective potential energy along each of these pathways can be modelled with a spherical potential well with an appropriate depth and width. The depth and width of each of the spherical potentials 
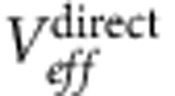
 and 
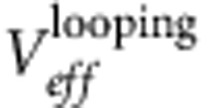
 are different for each pathway so that each one supports a different number of bound states *n*^direct^ and *n*^loop^, respectively. In the ultracold limit, the scattering phase shift associated with each pathway approaches a different integral multiple of *π*: *n*^direct^ *π* and *n*^loop^ *π*, and the phase difference Δ that appears in the interference term (when the direct and looping scattering amplitudes are combined to construct the total amplitude) is also an integral multiple of *π*: Δ=*mπ* where *m*=*n*^loop^−*n*^direct^. Furthermore, if the integer *m* is even (odd) then cos Δ=+1(−1) and *f*^NGP^ is non-zero (zero) and *f*^GP^ is zero (non-zero). Thus, in the ultracold regime, the effects of the GP can be large, essentially turning off or on the reactivity. In real molecular collisions, the phase quantization is not exact and the magnitudes of the direct and looping contributions are not identical so that the dynamic range is less than that described in the model above. However, the dynamic range of the GP effect can be dramatic in real ultracold reactions as we will now demonstrate for O+OH where the reactivity is suppressed or enhanced by nearly two orders of magnitude.

### Ultracold reaction rates for O+OH→H+O_2_

**Figure 3:**
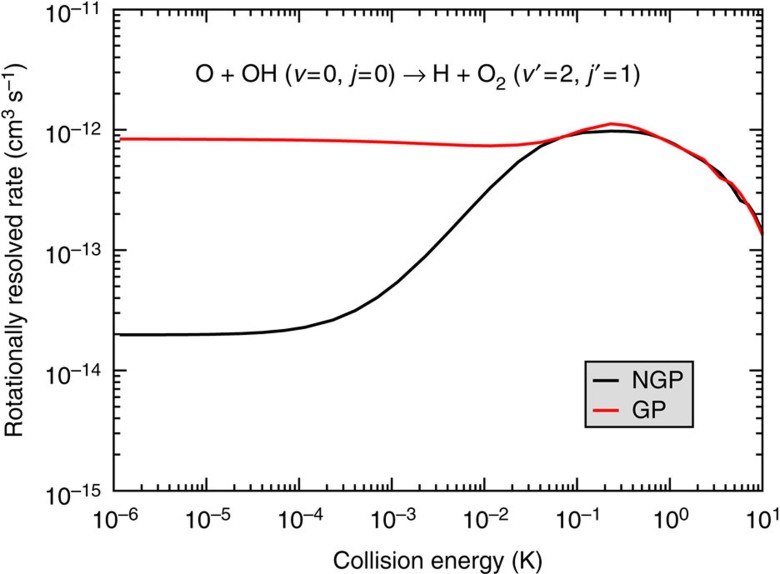
Geometric phase effects on a rotationally resolved reaction rate. The rotationally resolved O+OH (*v*=0, *j*=0)→H+O_2_ (*v*′=2, *j*′=1) reaction rate is plotted as a function of collision energy summed over all values of total angular momentum between *J*=0 and 3. Owing to constructive interference between the direct and looping pathways (see Fig. 2a), the ultracold (1 μK) reaction rate computed with the geometric phase (red) is dramatically enhanced relative to the ultracold rate computed without the geometric phase (black).

The effective Schrödinger equation for the nuclear motion is solved using an accurate time-independent quantum reactive scattering methodology that includes the GP (see Methods for more details)^11^,^12^,^46^,^47^,^48^,^49^. Figure 3 plots the computed reaction rate for O+OH (*v*=0, *j*=0)→H+O_2_ (*v*′=2, *j*′=1) as a function of collision energy summed over total angular momentum *J*=0–3. The red and black curves correspond to the calculations with (GP) and without (NGP) the GP, respectively. The ultracold energy regime where only s-wave scattering (that is, *J*=0) contributes to the reaction rate occurs at energies below 100 μK. Higher partial waves contribute for collision energies above 100 μK. The four values of total angular momentum *J* included in these calculations give converged reaction rates for collision energies up to ∼0.2 K (see Supplementary Fig. 1, which plots the individual contributions from each value of *J* and the associated Supplementary Discussion). Figure 3 shows that at ultracold collision energies (1 μK) the reaction rate that includes the GP (8.4 × 10^−13^ cm^3^ s^−1^) is over 40 times larger than the reaction rate that does not include the GP (2 × 10^−14^ cm^3^ s^−1^). As discussed above, the increased reaction rate for the GP calculations is due to the positive sign on the interference term between the direct and looping contributions to the scattering amplitude (see also Supplementary Fig. 2). A positive (negative) sign on the interference term leads to maximum constructive (destructive) interference between the direct and looping contributions to the total scattering amplitude (see Fig. 2). This particular ultracold reaction corresponds to the idealized case discussed above where cosΔ=−1 and 
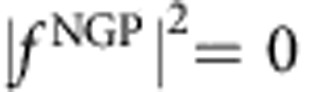
 and 
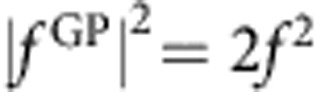
. As the collision energy increases, the *J*=1 contribution to the reaction rate becomes significant leading to a rapid increase in the NGP reactivity (black curve) starting at ∼100 μK. The GP (red) and NGP (black) reaction rates become nearly identical in magnitude once the collision energy reaches 0.05 K where several values of non-zero *J* contribute. The increased reactivity for the NGP case and *J*=1 is due to the presence of additional hyperspherical angular basis functions with symmetric (even) exchange symmetry with respect to the identical oxygen nuclei. For *J*=0 the Wigner D-function is symmetric and only hyperspherical angular basis functions with antisymmetric (odd) exchange symmetry are physically allowed^50^. For *J*>0 the Wigner D-functions contain symmetric and antisymmetric components that require the multiplication by *both* even and odd hyperspherical angular basis functions^14^,^47^. The presence of the additional even symmetry states alters the relative number of bound states (*m*) in the effective spherical well potentials along the direct and looping pathways (see Fig. 2). The change in *m* in turn alters the relative sign on the interference term giving constructive instead of destructive interference for the NGP case and *J*=1 (see Supplementary Fig. 2 and the associated Supplementary Discussion).

**Figure 4:**
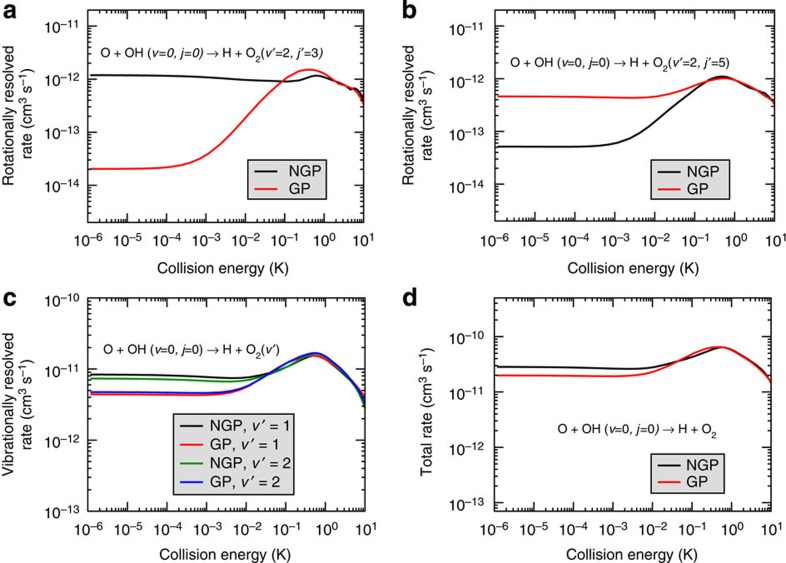
Geometric phase effects on other rotationally and vibrationally resolved reaction rates and the total rate. The rotationally resolved O+OH (*v*=0, *j*=0)→H+O_2_ (*v*′=2, *j*′) reaction rates for *j*′=3 and 5 are plotted as a function of the collision energy in **a**,**b**, respectively. The rates are summed over all values of total angular momentum between *J*=0 and 3. The ultracold (1 μK) reaction rate for *j*′=3 computed without the geometric phase (black) is dramatically enhanced relative to the rate computed with the geometric phase (red). In contrast, for *j*′=5 it is the rate computed with the geometric phase (red) that is enhanced. The vibrationally resolved reaction rate is plotted in **c** for *v*′=1 and 2 summed over all open product rotational levels *j*′. For both vibrational states, the rates computed with the geometric phase (red for *v*′=1 and blue for *v*′=2) lie below the rates computed without the geometric phase (black for *v*′=1 and green for *v*′=2). The total reaction rate is plotted in **d** summed over all open product vibrational and rotational states of O_2_ (*v*′, *j*′). Effects of the geometric phase remain even after the sums that results in a decreased ultracold reaction rate (red) relative to the total rate computed without the geometric phase (black).

Figure 4 shows rovibrationally resolved and total reaction rates for the O+OH (*v*=0, *j*=0) reaction. Reaction rates for *j*′=3 and 5 within the *v*′=2 vibrational level are plotted, respectively, in Fig. 4a,b. In contrast to the *j*′=1 result shown in Fig. 3a, the GP result (2 × 10^−14^ cm^3^ s^−1^) for *j*′=3 at 1 μK is ∼60 times *smaller* than the corresponding NGP result (1.2 × 10^−12^ cm^3^ s^−1^). On the other hand, the GP result (4.6 × 10^−13^ cm^3^ s^−1^) for *j*′=5 (Fig. 4b) is about nine times *larger* than the NGP result (5.2 × 10^−14^ cm^3^ s^−1^) at 1 μK. These findings show that both the GP and NGP ultracold reaction rates can be either dramatically enhanced or suppressed depending on the particular product rotational state involved. As discussed above, different product (and reactant) states give rise to different effective spherical well potentials along the direct and looping pathways. Thus, the relative phase shift (*mπ*) and the sign of the interference term between the direct and looping contributions to the scattering amplitude is sensitive to the states involved. Large differences between the GP and NGP ultracold reaction rates are also observed for *j*′>5 and other vibrational states *v*′. For example, vibrationally resolved reaction rates for *v*′=1 and 2 summed over all open product rotational levels *j*′ are plotted in Fig. 4c. In both cases differences remain between the ultracold reaction rates computed with and without the GP even after summing over many values of *j*′. For *v*′=1 and 2 the ultracold reaction rate computed with the GP is 1.9 and 1.6 times smaller than the NGP rate, respectively. Similar results are also seen for *v*′=0 and 3 (not shown) where the ultracold reaction rate computed with the GP is 1.1 and 1.3 times smaller than the NGP rate, respectively. All of the vibrationally resolved ultracold reaction rates (summed over all *j*′) are decreased when the GP is included. The total reaction rate summed over all open vibrational *v*′ and rotational *j*′ product states is plotted in Fig. 4d. The total ultracold reaction rate computed with the GP is 1.4 times smaller than the NGP rate.

## Discussion

The answer to the previously posed question ‘Can GP effects become enhanced at ultracold collision energies?' is a definite ‘yes'. In fact, the ultracold reaction rate is often controlled by the GP. As demonstrated for the O+OH→H+O_2_ reaction, the rotationally resolved reaction rates can be enhanced or suppressed by nearly two orders of magnitude when the GP is included in the theoretical calculations. The vibrationally resolved and total reaction rates can also be altered by the GP (by factors of nearly two). The enhanced effects of the GP reported here are general and originate from the unique features of the ultracold and cold energy regimes where only one or a few partial waves contribute to the scattering process. In this energy regime the scattering is essentially isotropic and results in an effective quantization of the relative phase shift between the direct and looping contributions to the total scattering amplitude (see Supplementary Fig. 3 and the associated Supplementary Discussion). The phase shift quantization and isotropic scattering results in maximum constructive or destructive interference between the direct and looping pathways. Since the GP changes the sign of the interference term between the direct and looping pathways, it controls whether the interference is constructive or destructive. The effects of the GP on all of the O+OH→H+O_2_ reaction rates can be explained using this simple interference mechanism (see Fig. 2 and Supplementary Figs 2 and 3). The enhancement or suppression of the ultracold reactivity due to the GP is a general result independent of the details of the PES. Of course which particular product states are enhanced or suppressed and the magnitude of the effect does depend on the PES. The GP must be included in the theoretical calculations of ultracold reaction rates for which the molecular Born–Oppenheimer electronic PES contains a CI and its encirclement is energetically favourable. Furthermore, the analysis reported here for the direct and looping contributions to the scattering amplitude also holds for reactive processes that involve interference between reactive and non-reactive (inelastic) pathways, such as H+HD→H+HD (refs 5, 9). Other experimentally relevant ultracold molecular systems are also known to contain CIs, such as Li+Li_2_ (ref. 51). An intriguing future application based on the results presented here might include using external electric and magnetic fields to tune through a zero energy resonance and alter the number of bound states (and hence the relative phase shift) for either the direct or looping pathways. The energy differences between the highest-lying bound states of HO_2_ are very small (≈5 K) and the highest bound state lies only 21 K below the dissociation threshold^13^. The Stark energy shift for OH in a typical laboratory external DC electric field of 100 kV cm^−1^ is ≈4 K, which is of comparable magnitude. Thus, it may be possible to use external fields to alter the relative number of bound states between the direct and looping pathways. This technique could provide experimentalists with a switch for turning the reactivity on or off.

## Methods

### Computational approach

The three-body quantum reactive scattering problem for a given molecular Born–Oppenheimer electronic PES is solved using a three-dimensional time-independent scattering method based on hyperspherical coordinates^46^. The methodology has been generalized to include the GP[11] and non-zero total angular momentum *J* (ref. 47), and has been parallelized to run efficiently on large supercomputers^14^,^15^. It has been applied to several reactive systems over a wide range of collision energies from ultracold to thermal. Details of the methodology are given elsewhere^11^,^46^,^47^,^48^; therefore, a brief summary will be presented here. The method is numerically exact (that is, no dynamical approximations are made) and well suited for accurately treating ultracold chemistry^49^. In the present work, the *ab initio* based DIMKP[42] PES with an improved long-range interaction^36^ is used for the lowest lying ^2^*A*′′ electronic state of HO_2_. The DIMKP PES accurately treats the CIs and includes a long-range potential that was absent in the *ab initio* XXZLG PES for this system^52^. Recently, a CHIPR PES has been developed, which is based on the same *ab initio* data set as the XXZLG PES but includes a long-range potential^39^,^40^. We refer the reader to previous work for detailed comparisons of the scattering results using these PESs^35^,^36^,^37^,^38^,^39^,^40^.

Smith–Johnson symmetrized hyperspherical coordinates are used in the interaction region where the three atoms are in close proximity (that is, for small hyperradius *ρ*)^46^,^47^. For larger hyperradius where the reactant (O+OH) and product (H+O_2_) channels become well defined, a properly symmetrized set of Fock–Delves hyperspherical coordinates are used (one for each channel)^48^. The hyperradius *ρ* is discretized into a set of sectors spanning the range from small to large *ρ*. The three-body Hamiltonian is diagonalized at each fixed value of *ρ* to obtain a set of 5D angular wave functions. The 5D angular solutions are independent of the collision energy so only have to be computed once for each value of total angular momentum *J* and inversion parity. The 5D angular solutions form the basis set for the coupled-channel equations in *ρ* and are used to compute a set of potential coupling matrices within each sector and the overlap matrices between the different 5D solutions at the boundaries of each sector. The coupled-channel equations are solved for a given collision energy using a log-derivative propagator method from small to large *ρ*. Finally, the asymptotic boundary conditions are applied at large *ρ* to compute the scattering *S* matrix from which the cross-sections and reaction rates can be computed^46^. Detailed convergence studies, basis sets and other parameters used for the O+OH reaction are reported elsewhere^35^,^36^,^37^,^38^.

## Additional information

**How to cite this article:** Kendrick, B. K. *et al*. The geometric phase controls ultracold chemistry. *Nat. Commun.* 6:7918 doi: 10.1038/ncomms8918 (2015).

## Supplementary Material

Supplementary Information Supplementary Figures 1-3 and Supplementary Discussion
