# Supplementary material for: The geometric phase controls ultracold chemistry
Source: Nat Commun. 2015 Jul 30;6:7918. doi: 10.1038/ncomms8918 (PMC4532881; doi:10.1038/ncomms8918)
Supplement: Supplementary Information — Supplementary Figures 1-3 and Supplementary Discussion [file ncomms8918-s1.pdf]

Supplementary Information

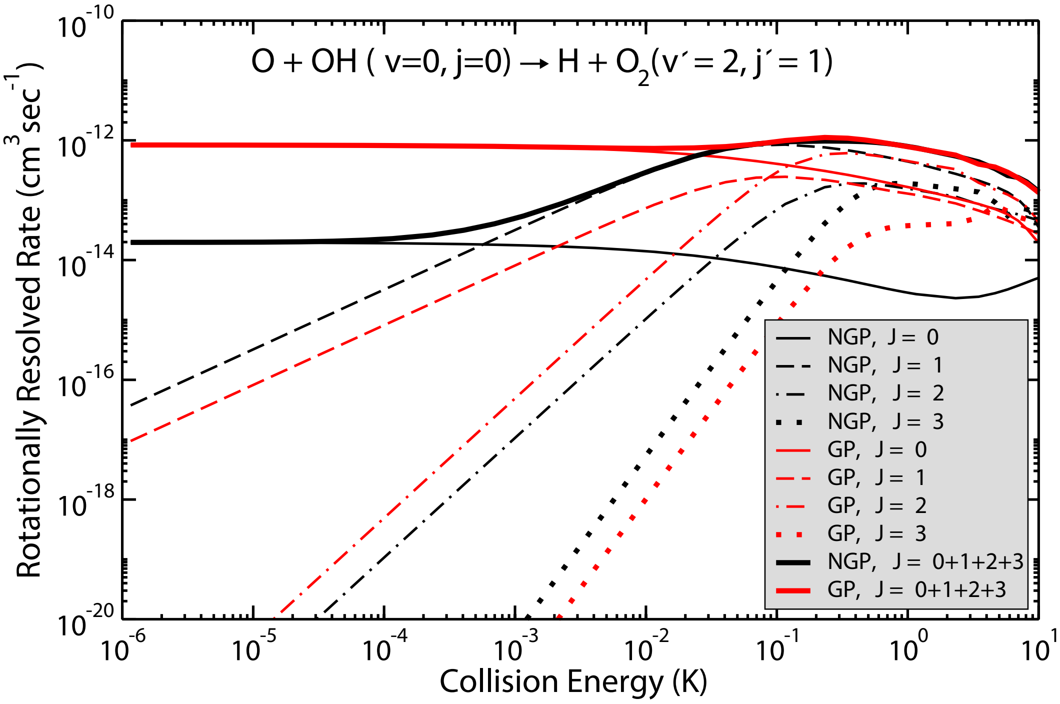

Supplementary Figure 1. | **Total angular momentum contributions to the reaction rate.** The individual contributions to the reaction rate for O + OH(v = 0, j = 0) → H + O_2(v' = 2, j' = 1) from each value of total angular momentum J = 0 - 3 are plotted (see legend). For each value of J, the rates computed with (red) and without (black) the geometric phase are also plotted. The GP and NGP reaction rates summed over all values of J = 0 - 3 are shown as thick red and black curves, respectively (see Supplementary Discussion).

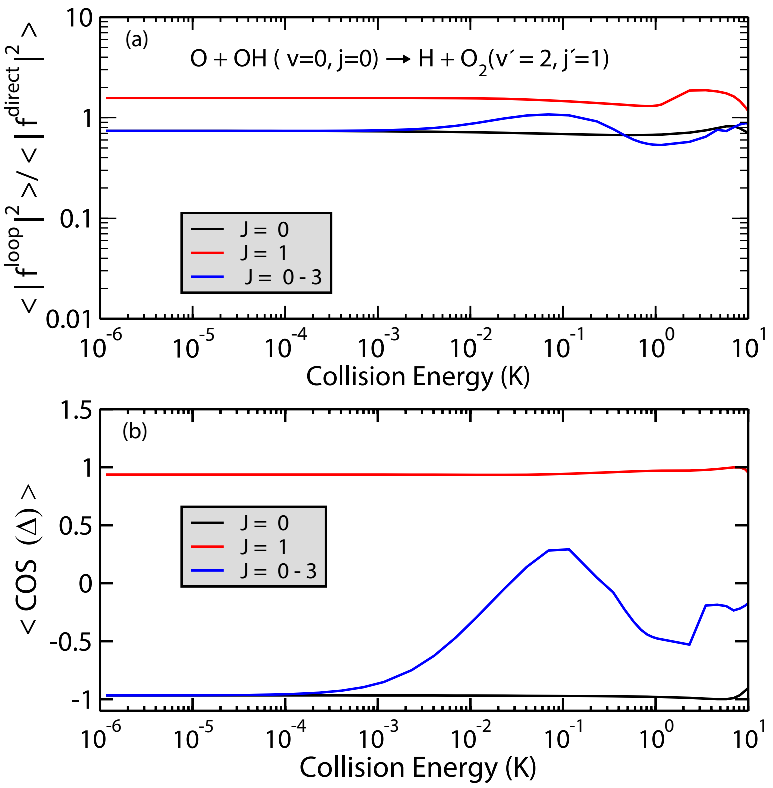

Supplementary Figure 2. | **Relative magnitudes and phases of the looping and direct scattering amplitudes.** In panel (a), the ratio of the average square modulus of the looping and direct contributions to the total scattering amplitude is plotted as a function of the collision energy for the O + OH($v = 0$, $j = 0$) $\rightarrow$ H + O_2($v' = 2$, $j' = 1$) reaction. The black and red curves correspond to single values of $J = 0$ and 1, respectively. The blue curve corresponds to the sum over all values of J between 0 and 3. Panel (b) plots the average value of $\cos\Delta$ as a function of the collision energy using the same color designations as in panel (a) (see Supplementary Discussion).

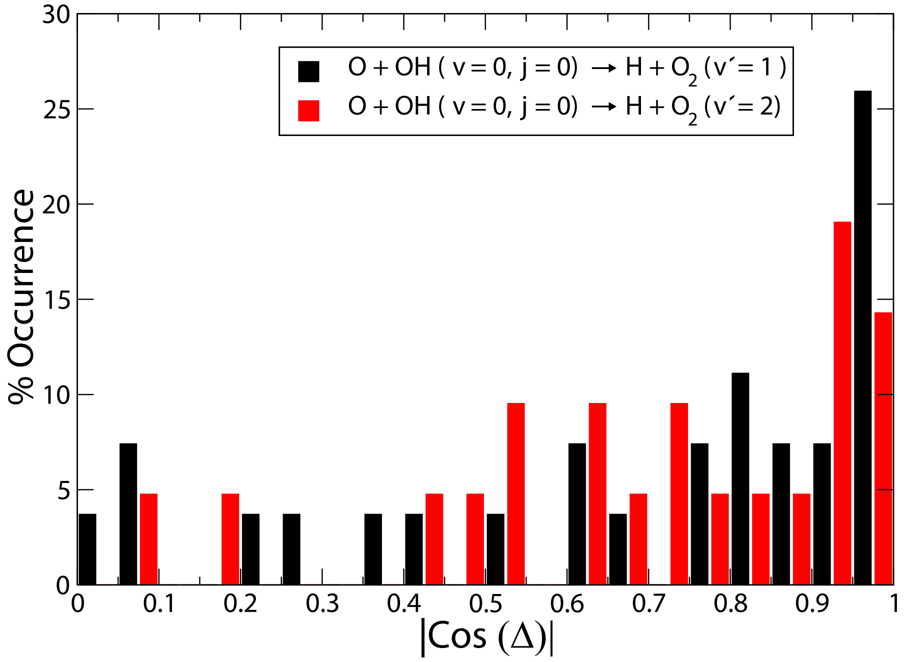

Supplementary Figure 3. | **Distribution of relative phases between the looping and direct scattering amplitudes.** The percentages of the total number of open $O_2(v' = 1,\, j')$ (black) and $O_2(v' = 2,\, j')$ (red) product states with values of $|\cos\Delta|$ lying in a particular interval of $|\cos\Delta|$ are plotted at a collision energy of $1\,\mu$K. The interval spacing bin size along the x-axis is 0.05. The distribution is clearly biased towards the right edge (i.e., $|\cos\Delta| \approx 1$) due to the quantization of the scattering phase shift in the ultracold limit (see Supplementary Discussion).

Supplementary Discussion. The individual contributions from each value of total angular momentum $J = 0$ - 3 to the O + OH($v = 0$, $j = 0$) $\rightarrow$ H + O_2($v' = 2$, $j' = 1$) reaction rate are plotted in Supplementary Fig. 1 as a function of collision energy. The rates for each value of J are well converged over the entire energy range. The red and black curves correspond to calculations with and without the geometric phase (GP), respectively. The thick solid curves include contributions from all four values of total angular momentum and are well converged with respect to the sum over J for collision energies up to approximately 0.2 K. The thin solid curves correspond to $J = 0$, the long dashed curves are $J = 1^-$, the dashed-dotted curves are $J = 2^+$, and the diamonds are $J = 3^-$ (the $\pm$ here indicates inversion parity). At low collision energies (i.e., < 0.1 K) only a few partial waves contribute to the reaction rate. Due to the angular momentum barrier in the entrance channel which increases with increasing J, the contributions to the reaction rate from higher values of J decrease rapidly with decreasing collision energy. In the ultracold limit (1 μK) only the contribution from $J = 0$ is significant (thin solid curves). For each value of J the red and black curves alternate between which one is larger. For other product states, the geometric phase (GP) and no geometric phase (NGP) contributions vary for different values of J but they do not always strictly alternate with even and odd J as they do in Supplementary Fig. 1. In the ultracold and cold energy regime, whether or not the GP or NGP contribution is largest for a given J depends on the relative number of bound states m in the effective spherical well potentials associated with the direct and looping pathways around the CI (see also Supplementary Fig. 2 and discussion below).

Supplementary Figure 2a plots the ratio $\langle |f^{\text{loop}}|^2 \rangle / \langle |f^{\text{direct}}|^2 \rangle$ as a function of collision energy for the O + OH($v = 0$, $j = 0$) $\rightarrow$ H + O_2($v' = 2$, $j' = 1$) reaction (where $\langle\,\rangle$ denotes the average value defined as $\langle\,\rangle = (1/\pi) \int_0^\pi \mathrm{d}\theta$ and θ is the scattering angle). The square modulus of the scattering amplitudes are summed over the quantum numbers $m_{j'} = +1$, 0, and -1 for the product O_2 diatomic rotational state $j' = 1$. The black curve corresponds to $J = 0$, the red curve is $J = 1^-$ and the blue curve includes all values of $J = 0$ - 3. For collision energies < 1 K the ratio in all cases is close to unity. Thus, the direct and looping contributions to the scattering amplitude are comparable in magnitude and the square modulus of the total scattering amplitude for the NGP and GP cases can be approximated as $|\,f^{\text{NGP}}|^2 = f^2\,(1 + \cos\Delta)$ and $|\,f^{\text{GP}}|^2 = f^2\,(1 - \cos\Delta)$, respectively. If the magnitude of $|\cos\Delta| \approx 1$ then maximum constructive or destructive interference occurs

and, depending upon the sign of $\cos\Delta$, either the NGP or GP scattering amplitude will be larger than the other. The phase Δ is the relative phase shift between the direct and looping scattering amplitudes.

Supplementary Figure 2b plots the average $\langle\cos\Delta\rangle = (1/\pi)\int_0^\pi \cos\Delta\, d\theta$ as a function of collision energy for $J = 0$ (black), $J = 1^-$ (red), and summed over all $J = 0$ - 3 (blue). Only the $\cos\Delta$ for $|m_{j'}| = 1$ are plotted (the curves for $m_{j'} = 0$ are similar). In the ultracold limit ($1\,\mu$K) only $J = 0$ contributes to the reaction rate and from Supplementary Fig. 2b we see that for $J = 0$, $\langle\cos\Delta\rangle \approx -1$. The corresponding square modulus of the scattering amplitudes for $J = 0$ are $|\, f^{\rm NGP}|^2 \approx 0$ and $|\, f^{\rm GP}|^2 \approx 2\, f^2$ which explains the very large difference between the GP (red) and NGP (black) ultracold reaction rates observed in Fig. 3 of the article and Supplementary Fig. 1. As the collision energy increases above $100\,\mu$K, contributions to the reaction rate from $J = 1^-$ become important. Supplementary Figure 2b shows that for $J = 1^-$ and small collision energies, $\langle\cos\Delta\rangle \approx +1$. Thus, in this case $|\, f^{\rm NGP}|^2 \approx 2\, f^2$ and $|\, f^{\rm GP}|^2 \approx 0$ which explains why the black dashed curve is larger than the red one in Supplementary Fig. 1. As more partial waves contribute to the scattering at higher collision energies, $\langle\cos\Delta\rangle$ decreases in magnitude and tends to oscillate about zero (the blue curve in Supplementary Fig. 2b). As $\langle\cos\Delta\rangle$ decreases in magnitude, the differences between the $|\, f^{\rm NGP}|^2$ and $|\, f^{\rm GP}|^2$ become smaller which explains why the GP (thick solid red curve) and NGP (thick solid black curve) results are nearly identical at higher collision energies (0.05 K) in Supplementary Fig. 1. Similar behavior occurs for other product rotational states. For example, for $j' = 3$ the situation is reversed and for $J = 0$ and ultracold collision energies $\langle\cos\Delta\rangle \approx +1$ which implies that $|\, f^{\rm NGP}|^2 \approx 2\, f^2$ and $|\, f^{\rm GP}|^2 \approx 0$. This explains why the NGP ultracold reaction rate (black curve) is much larger than the GP one (red curve) in Fig. 4a in the article. The situation for $j' = 5$ is similar to that for $j' = 1$. For some higher lying rotational states, such as $j' = 7$, 13, 19, and 21, either $\langle\cos\Delta\rangle$ is small and/or the ratio $\langle|f^{\rm loop}|^2\rangle/\langle|f^{\rm direct}|^2\rangle$ is $\ll 1$ or $\gg 1$. For these product rotational states there is little difference between the ultracold $|\, f^{\rm NGP}|^2$ and $|\, f^{\rm GP}|^2$.

Supplementary Figure 3 plots a histogram representing the percentages of the total number of open O_2 product states with an absolute value of $\cos\Delta$ which lies within a given interval of $|\cos\Delta|$ for a collision energy of $1\,\mu$K. The intervals or bins in $|\cos\Delta|$ are equally spaced along the x axis with size 0.05. Two histograms are plotted, one for $O_2(v' = 1, j')$ (black) and another for $O_2(v' = 2, j')$ (red). The distribution is significantly biased

towards the right edge where $|\cos\Delta| \approx 1$ due to the quantization of the scattering phase shift which occurs at ultracold collision energies (see Fig. 2 in article). Approximately 60% of the $v' = 1$ (black) product states have $|\cos\Delta| > 0.75$ and half of these lie in the range $|\cos\Delta| > 0.9$ (i.e., the relative phase shift between the looping and direct pathways is close to an integer multiple of π). Similarly, approximately 50% of the $v' = 2$ (red) product states have $|\cos\Delta| > 0.75$ and 70% of these lie in the range $|\cos\Delta| > 0.9$.
